# Development of Biosimilar Aflibercept SDZ-AFL

**DOI:** 10.1016/j.curtheres.2025.100812

**Published:** 2025-08-31

**Authors:** Francis Dodeller, Peter Alliger, Jens Heyn, Dragan Urosevic, Lisa Allmannsberger, Cornelia Wersig, Rufino Silva

**Affiliations:** 1 HEXAL AG, Holzkirchen, Germany; 2 Sandoz GmbH, Kundl, Austria; 3 Sandoz AG, Basel, Switzerland; 4 Faculty of Medicine, University of Coimbra, Coimbra, Portugal; 5 Ophthalmology Department, ULS de Coimbra, Portugal; 6 Association for Innovation and Biomedical Research on Light and Image (AIBILI), Coimbra, Portugal

**Keywords:** Aflibercept, Biosimilar, Neovascular age-related macular degeneration, Retinal disease, SOK583A1

## Abstract

**Purpose:**

Aflibercept is a recombinant fusion protein that binds with high affinity to vascular endothelial growth factor A (VEGF-A), and other growth factors, reducing pathological neovascularization and abnormal vascular permeability. Aflibercept is approved as a first-line treatment for several retinal diseases, including neovascular age-related macular degeneration (nAMD); however, the high costs of these biologic agents can impede patient access. In 2024, Sandoz biosimilar aflibercept (SDZ-AFL; SOK583A1, Enzeevu/Afqlir) was approved by the US Food and Drug Administration and the European Medicines Agency as a biosimilar of reference aflibercept (Ref-AFL; Eylea, a trademark of Bayer AG and in the US of Regeneron Pharmaceuticals, Inc) based on a comprehensive package of data.

**Methods:**

This narrative review summarizes the totality of evidence demonstrating biosimilarity between SDZ-AFL and Ref-AFL, including physicochemical and biological characterization data from analytical in vitro studies, results of an ocular pharmacokinetic (PK) study in rabbits, and clinical efficacy, safety, immunogenicity, and systemic PK data from Phase III clinical studies.

**Results:**

Analytical evaluation demonstrated that SDZ-AFL has structural homology with Ref-AFL, including identical amino acid sequences, indistinguishable higher-order structures, and highly similar levels of structural variants. SDZ-AFL and Ref-AFL also demonstrated highly similar in vitro biologic activities*,* including target binding affinity, and potency in terms of neutralization of VEGF-A. In a single-dose ocular PK study in rabbits, vitreal exposure was comparable between SDZ-AFL and Ref-AFL after intravitreal administration. A 52-week Phase III clinical study (Mylight) evaluated the efficacy, safety, immunogenicity, and systemic PK of SDZ-AFL and Ref-AFL in 484 participants with nAMD. The primary endpoint was mean change from baseline to week 8 in best corrected visual acuity (BCVA): the difference between the SDZ-AFL and Ref-AFL groups in this endpoint was –0.3 letters (90% CI −1.5 to 1.0), which met predefined bioequivalence margins. Secondary efficacy endpoints, including changes from baseline in BCVA and central subfield foveal thickness over the whole 52-week study, were also similar for SDZ-AFL and Ref-AFL. Two subsequent “in-use” studies confirmed the safe use of SDZ-AFL provided in either a vial kit or a prefilled syringe.

**Conclusion:**

This comprehensive totality of evidence has established biosimilarity between SDZ-AFL and Ref-AFL based on comparable physicochemical and biological characteristics, as well as similarity in clinical efficacy, safety, and immunogenicity. The introduction of aflibercept biosimilars to the market is anticipated to reduce barriers to access, potentially increasing the number of appropriate patients with retinal diseases benefiting from this biologic therapy.

## Introduction

Aflibercept is a gold standard treatment for retinal diseases, such as neovascular age-related macular degeneration (nAMD),^1^ based on evidence from randomized clinical trials and routine clinical practice.^2^, ^3^, ^4^ Reference aflibercept is approved in the European Union (EU) and the United States (US) for treating adults with nAMD, macular edema following retinal vein occlusion (RVO), diabetic macular edema (DME), diabetic retinopathy (DR, US only), and myopic choroidal neovascularization (mCNV, EU only).^5^^,^^6^ It is approved in the US and EU for use in newborn infants to treat retinopathy of prematurity.^5^^,^^6^ Through inhibition of vascular endothelial growth factor (VEGF)-A, (VEGF)-B, and placental growth factor (PIGF), aflibercept reduces aberrant angiogenesis in the choroid, increasing vessel stability.^1^^,^^7^, ^8^, ^9^

Retinal and choroidal vascular diseases, such as DR, RVO, and nAMD, are a highly prevalent cause of moderate-to-severe vision loss: according to the World Health Organization, DR and nAMD account for 11.9 million cases of preventable vision loss globally.^10^ Biologic anti-VEGF agents can improve the visual acuity of people with retinal and choroidal vascular diseases and are an important therapy for several of these diseases, including nAMD and DR.^11^ However, the high costs of these biologic agents can provide a significant financial burden to patients and healthcare systems, creating a barrier to patient access and potentially leading to treatment discontinuation or non-adherence, ultimately worsening patient outcomes.^12^

With an ever-increasing prevalence of retinal and choroidal diseases projected in the coming years,^13^, ^14^, ^15^ the global demand for these costly anti-VEGF agents is also anticipated to rise. Biosimilar medicines, defined by the US Food and Drug Administration (FDA) as biological agents without any clinically meaningful differences in terms of safety, purity or potency vs another FDA-approved biologic agent (the reference medicine),^16^ represent cost-effective therapeutic alternatives to reference biologic medicines.^17^^,^^18^ The introduction of anti-VEGF biosimilar medicines will be paramount to mitigate the growing financial burden of these treatments as patents for anti-VEGF biologics approved for retinal diseases begin to expire.^16^^,^^17^ Biosimilars of approved anti-VEGF agents to treat retinal diseases started to enter the EU and US markets in 2021, with the first ranibizumab biosimilar available in the EU in August 2021 and in the US in July 2022.^19^^,^^20^ Since then, several other ranibizumab biosimilars have been marketed in the EU and US.^21^^,^^22^

Following approval, additional efficacy and safety data for intravitreal ranibizumab biosimilars have been generated from multiple real-world and observational studies without demonstrating any new safety signals.^23^, ^24^, ^25^, ^26^, ^27^, ^28^ In addition, cost-effectiveness modelling performed in Japan suggests that biosimilar ranibizumab is a cost-saving option compared with reference ranibizumab and reference aflibercept in the treatment of patients with nAMD.^29^^,^^30^

With the expiry of regulatory exclusivity for reference aflibercept (Ref-AFL; Eylea, a registered trademark of Bayer AG, and of Regeneron Pharmaceuticals Inc in the US) on the horizon, the European Medicines Agency (EMA) and the US Food and Drug Administration (FDA) have approved several aflibercept biosimilars, with first approvals in September 2023 (EU) and May 2024 (US).^31^ The FDA approved Sandoz aflibercept (SDZ-AFL) in August 2024 under the brand name Enzeevu^32^ and the European Commission approved it in November 2024 (Afqlir).^33^

**Figure 1:**
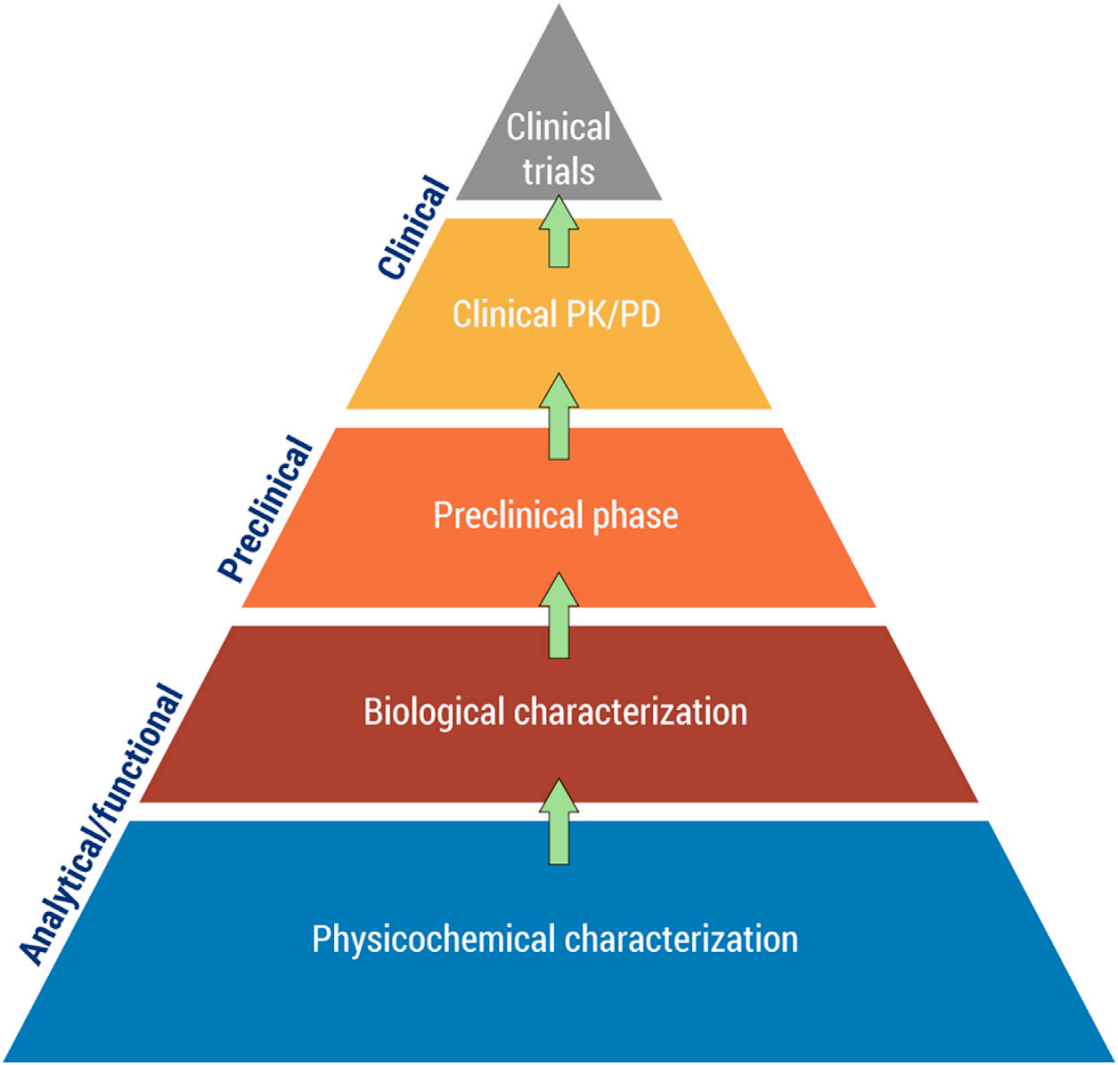
Totality-of-the-evidence approach for the development of biosimilars. PD = pharmacodynamics; PK = pharmacokinetics.

In this review, we summarize the development of SDZ-AFL, following the biosimilar concept, which is based on the totality of evidence.^34^ For biosimilar approval, the regulators request from the applicant a comprehensive set of analytical, functional, pharmacokinetic (PK)/pharmacodynamic (PD), and clinical data to demonstrate the bioequivalence of the biosimilar to its reference biologic (Figure 1). As the base of the evidence pyramid, the comprehensive structural and functional in vitro analyses of biosimilars contrast with the development of novel medicines, which must demonstrate efficacy and safety in clinical Phases I and II and in large pivotal Phase III trials for each indication.

Here, we provide an overview of the totality of evidence demonstrating biosimilarity between SDZ-AFL and Ref-AFL, including physicochemical and biological characterization data from analytical in vitro studies, non-clinical PK data from an in vivo study, and clinical efficacy, safety, immunogenicity, and PK data from a Phase III clinical study in patients with nAMD.

## Methods

Reference aflibercept from the US and EU was purchased through local distributors and stored and handled according to the manufacturer’s instructions.

### Analytical and Functional Characterization

The structural similarity between SDZ-AFL and Ref-AFL was assessed using multiple validated analytical methods to ensure thoroughness, reliability, and reproducibility. The testing plan is presented in Table 1. The correctness of the amino acid sequence of SDZ-AFL was confirmed at the protein, peptide, and amino acid levels. For this purpose, orthogonal analytical methods were used, including mass analysis of intact aflibercept and its subunits and amino acid sequencing by liquid chromatography–tandem mass spectrometry (LC-MS/MS) peptide mapping. Correct formation of disulfide bridges and completeness of linkage were assessed by non-reducing LC-MS peptide mapping (nrPepMap) and Ellman’s assay. Similarity in higher-order structure was confirmed using spectroscopic methods and assessment of thermal stability. Chromatographic, electrophoretic, and mass spectrometric methods analyzed post-translational modifications such as N-glycosylation, glycation, oxidation, deamidation, and N- and C-terminal variants.

**Table 1:** In vitro physicochemical and biological characterization of SDZ-AFL vs Ref-AFL.

Measurement: analysis of SDZ-AFL vs Ref-AFL	Methodology	Results
Structure		
Primary structure (protein detection and sequencing)	• Liquid chromatography coupled to peptide detection and sequencing by tandem mass spectrometry (LC-MS/MS)	Identical amino acid sequences
Higher-order structure	• Circular dichroism spectroscopy • Fourier-transform infrared spectroscopy (FT-IR) • Differential scanning fluorimetry (DSF)	Secondary and tertiary structures comparable
Cysteine chemistry	• Ellman’s assay • nrPepMap	Identical disulfide bridge profiles
Analysis of structural variants		
Glycosylation variants	• Normal phase HPLC after enzymatic release of N- glycans • Site-specific N-glycans by rPepMap	Similar N-glycan profiles and glycan site occupancy with minor differences in the abundance of some glycans
Charge variants	• Imaged capillary isoelectric focusing (iCE) • rPepMap	Minor differences in charge heterogeneity due to lower levels of Asn deamidation, C-terminal Lys, and higher prolineamide content
Size variants	• rPepMap, LC-MS/MS • Size exclusion chromatography (SEC) • Analytical ultracentrifugation • Non-reducing/reducing capillary electrophoresis with sodium dodecyl sulfate (nr/rCE-SDS)	Similar size variant profile with lower levels of dimers, aggregates, and fragments in SDZ-AFL
Functional characterization		
Binding to VEGF- A165, 189, 121, 110; Binding to VEGF-B167; Binding to PIGF-1 and PlGF-2	• ELISA and surface plasmon resonance	Similar in vitro biological activity with minor differences in binding
Potency assays	• VEGF-A165-induced HUVEC proliferation assay • VEGF-A165-induced VEGFR2 dimerization (cell based)	

HPLC = high-performance liquid chromatography; HUVEC = human umbilical vein endothelial cell; PIGF = placental growth factor; (n)rPepMap = (non-)reducing liquid chromatography–mass spectrometry peptide mapping; Ref-AFL =reference aflibercept; SDZ-AFL = Sandoz aflibercept; VEGF = vascular endothelial growth factor; VEGFR = VEGF receptor.

The analytical methods used for the in vitro physicochemical and biological characterization of SDZ-AFL are also listed in Table 1.

Target binding of SDZ-AFL and Ref-AFL to VEGF-A isoforms, VEGF-B, and PIGF-2 was assessed by ELISA and surface plasmon resonance. The bioactivity of SDZ-AFL and Ref-AFL was assessed in a proliferation assay using human umbilical vein endothelial cells to determine the neutralization of VEGF-A165-mediated cell proliferation; this was complemented by a cell-based VEGFR2 dimerization assay.

### Ocular PK Study in Rabbits

A single bilateral intravitreal injection (1.2 mg, 30 µL/eye) of SDZ-AFL or Ref-AFL was administered to 36 New Zealand white rabbits. Clinical observations, including body weight, food consumption, and ophthalmology evaluations, were performed daily for 1 week and then once weekly. Vitreous PK was assessed from vitreous humor collected from 3 animals per treatment group per time point at 1, 24, 48, 120, 216, and 360 hours postdose; blood for systemic PK and anti-drug antibody (ADA) evaluation was collected from all animals predose and from 3 animals per treatment group per time point at 1, 24, 48, 120, 216, and 360 hours post-dose. Total drug concentration (vitreous and serum) and ADA levels (serum) were measured using ELISA and electrochemiluminescence (ECL) assays, respectively. Ocular PK parameters were calculated by non-compartmental analysis based on the mean concentrations at each time point. All animal procedures followed the Animal Welfare Act Regulations (9 CFR 3). The study protocol was reviewed and approved by Labcorp and by the animal welfare offices at Labcorp and at Novartis/Sandoz.

### Clinical Trial Data for SDZ-AFL

#### Phase III Efficacy, Safety, and Immunogenicity Study in Humans (Mylight Study)

The design and methods of the Phase III efficacy and safety study of SDZ-AFL versus Ref-AFL in 484 patients with nAMD have been described previously (Mylight; NCT04864834).^35^^,^^36^ In brief, anti-VEGF-naïve patients ≥50 years of age with nAMD with active lesions were randomized to receive intravitreal injections of SDZ-AFL or Ref-AFL for 52 weeks. The primary endpoint was mean change from baseline in best corrected visual acuity (BCVA) at week 8.^35^^,^^36^ Additionally, concentrations of free VEGF in plasma were quantified using an ECL assay in a subset of patients at week 48 (pre-dose, used as a baseline) and at week 52 (4 weeks post-dose, with a lower limit of quantification of 0.493 pg/mL).

#### In-Use Studies of SDZ-AFL Provided as a Vial Kit or Prefilled Syringe

Two open-label, single-arm, multicenter studies in patients with nAMD were designed to evaluate the safety of use of SDZ-AFL (40 mg/mL) provided in a vial kit (Study 303; NCT05282004)^37^ or prefilled syringe (Study 304; NCT05161806).^38^ Each of these studies evaluated a single intravitreal injection of SDZ-AFL (2 mg in 0.05 mL).^37^^,^^38^ The primary safety endpoint for both studies was the occurrence of ocular or non-ocular adverse events until the end of the study-reporting period (day 31, plus a 4-day window of tolerance).

## Results

### Analytical and Functional Characterization of SDZ-AFL

Structural similarity between SDZ-AFL and Ref-AFL was demonstrated via numerous parameters. The full amino acid sequence was identical for both products (Table 1). Similar secondary and tertiary structures and thermal stability were also confirmed, demonstrating the matching higher-order structures of SDZ-AFL and Ref-AFL **(**Table 1**)**.

**Figure 2:**
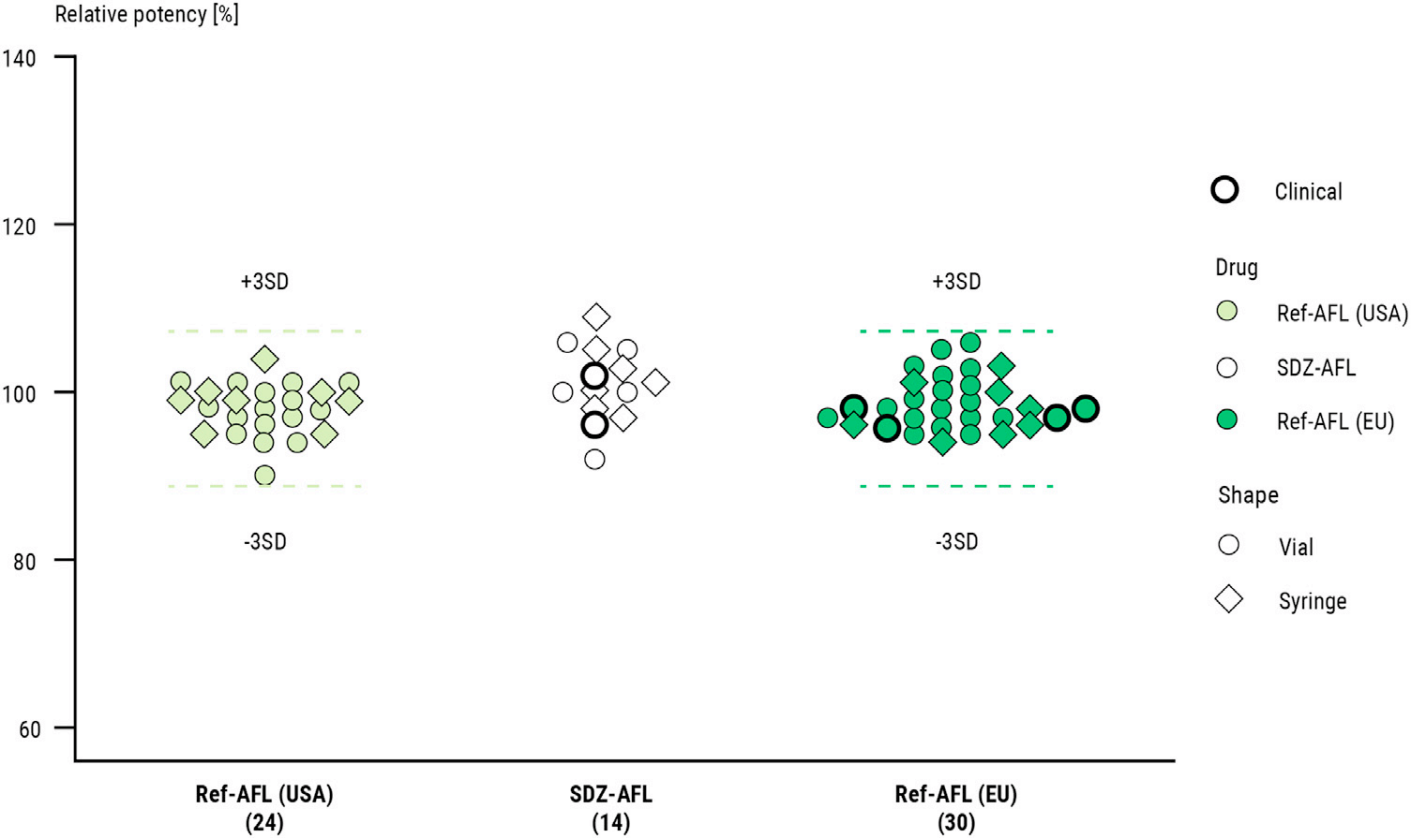
Comparison of the VEGF-A neutralizing potency of SDZ-AFL and Ref-AFL (EU- and US-sourced vials and prefilled syringes) in a cell-based HUVEC proliferation assay. HUVEC = human umbilical vein endothelial cell; Ref-AFL = reference aflibercept; SDZ-AFL = Sandoz aflibercept; VEGF = vascular endothelial growth factor.

Structural variants, glycosylation variants, and charge variants were shown to be highly similar between SDZ-AFL and Ref-AFL (Table 1). Biological characterization showed that SDZ-AFL and Ref-AFL have similar activity. The 2 products showed overall similar binding affinities to several VEGF-A isoforms, VEGF-B, and PIGF (Table 1). Minor differences in binding were observed for some variants, which were caused by the lower levels of impurities in SDZ-AFL (dimers, aggregates, fragments). The results from the human umbilical vein endothelial cell proliferation assay and the VEGFR-2 dimerization assay showed SDZ-AFL and Ref-AFL to have similar potency in terms of neutralization of VEGF-A165 (Figure 2, Table 1). A small but consistent increase in the potency of SDZ-AFL compared with Ref-AFL (3%–4% increase on average) was observed. This was determined to be due to lower levels of product-related impurities in SDZ-AFL than Ref-AFL. This minor difference in potency was not considered to be clinically relevant.

#### Ocular PK Study in Rabbits

**Figure 3:**
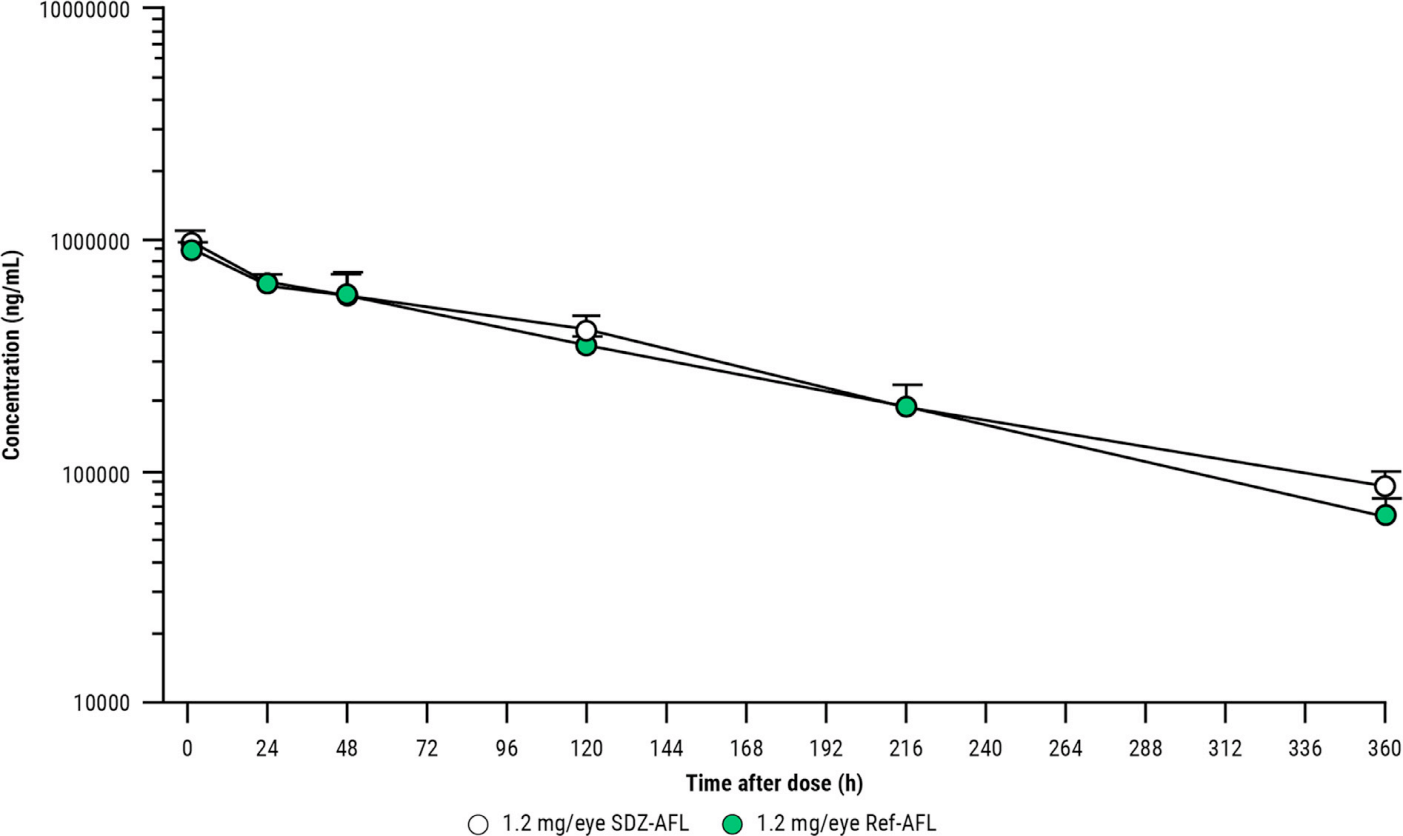
Mean (+SD) vitreal concentration of SDZ-AFL and Ref-AFL in rabbits over time. Ref-AFL = reference aflibercept; SDZ-AFL = Sandoz aflibercept.

Peak aflibercept concentrations in the vitreous were observed at the first sampling time point (1 hour) and subsequently decreased in a mono-exponential manner. Aflibercept concentrations in the vitreous and vitreous PK parameters were alike for SDZ-AFL and Ref-AFL (Figure 3), as was the exposure of aflibercept (AUC_0-t_) in the vitreous (117,000 h*µg/mL for SDZ-AFL and 111,000 h*µg/mL for Ref-AFL). Aflibercept was detected in the serum, with the maximum concentration observed at 120 hours post-dose (C_max_ 2280 ng/mL for SDZ-AFL and 2000 ng/mL for Ref-AFL). The systemic serum aflibercept concentrations were low overall—approximately 400-fold lower than those observed in the vitreous humor at C_max_—and comparable between SDZ-AFL and Ref-AFL. Two animals (one receiving each treatment) tested positive for ADAs without any apparent impact on vitreous or systemic PK (data not shown). No treatment-related differences in body weight, food consumption, or clinical observations were noted for any animal.

Mean (+SD) vitreal concentration of SDZ-AFL and Ref-AFL in rabbits over time (Figure 3). Ref-AFL = reference aflibercept; SDZ-AFL = Sandoz aflibercept.

### SDZ-AFL Clinical Trials

#### Phase III Efficacy, Safety, Immunogenicity, and PK/PD Study in Patients With nAMD

The findings of the Mylight study have been described previously.^35^^,^^36^ In brief, treatment groups (SDZ-AFL, *n* = 244, and Ref-AFL, *n* = 240) were well balanced with respect to demographics (age, sex, and geographic region) and baseline characteristics, including BCVA, CNV lesion size and type, central subfield foveal thickness (CSFT), and subretinal and intraretinal fluid.^36^

**Figure 4:**
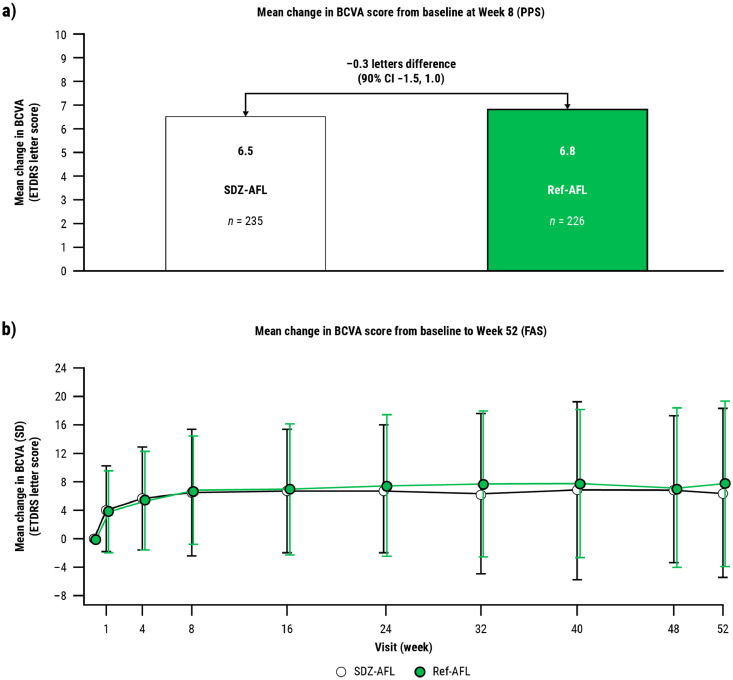
**(A)** Primary endpoint: Mean change in BCVA score from baseline to week 8; **(B)** Secondary endpoint: Mean change from baseline in BCVA at all visits up to week 52.^35^^,^^36^ BCVA = best corrected visual acuity; ETDRS = early treatment diabetic retinopathy study; FAS = full analysis set; PPS = per protocol set; Ref-AFL = reference aflibercept; SDZ-AFL = Sandoz aflibercept.

The primary endpoint was met: the difference between the SDZ-AFL and Ref-AFL groups in the mean change from baseline at week 8 in BCVA score was –0.3 letters (90% CI, −1.5 to 1.0) in the per-protocol set (PPS). This was within the predefined bioequivalence margins: the 95% CI for a difference in mean change within margins of ±3.5 letters (by the EMA) and the 90% CI for a difference in mean change within margins of ±3.0 letters (by the FDA) (Figure 4**A**).^36^

The secondary efficacy endpoint of mean change in BCVA score from baseline was equivalent in the 2 treatment groups at all time points up to week 52 (Figure 4**B**).^35^^,^^36^ Anatomical assessments demonstrated CSFT to be similar in the 2 groups at all time points up to week 52 and CNV lesion size to be similar at weeks 8 and 52.^36^ An additional efficacy analysis showed the proportion of patients with intraretinal fluid at week 52 to be similar: 23.9% in the SDZ-AFL group and 24.3% in the Ref-AFL group. Similarly, categories for BCVA maintenance or gains were comparable between the SDZ-AFL and Ref-AFL treatment groups: 16.7% of the SDZ-AFL group and 17.2% of the Ref-AFL group lost fewer than 15 letters, and 20.3% of the SDZ-AFL group and 23.8% of the Ref-AFL group gained 15 or more letters.^36^

The overall safety profile was comparable between both treatment groups in the Mylight study, including the incidence of ocular and non-ocular treatment-emergent adverse events (TEAEs), serious TEAEs, and TEAEs suspected to be treatment-related (Table 2).^36^ In terms of ocular adverse events, one patient experienced intraocular inflammation and another patient experienced retinal vasculitis, both in the Ref-AFL group. No cases of endophthalmitis or retinal vascular occlusion were observed during the study.

**Table 2:** Treatment-emergent adverse events in the Phase III Mylight study in nAMD.^31^

Category	SDZ-AFL (*n* = 244)	Ref-AFL (*n* = 240)
TEAE, *n* (%)	179 (73.4)	174 (72.5)
TEAE, suspected to be treatment-related, *n* (%)	6 (2.5)	7 (2.9)
SAE, *n* (%)	39 (16.0)	30 (12.5)
SAE, suspected to be treatment-related, *n* (%)	2 (0.8)	1 (0.4)
Ocular TEAE in study eye, procedure-related, *n* (%)	28 (11.5)	26 (10.8)
Ocular TEAE in study eye, treatment-related, *n* (%)	5 (2.0)	7 (2.9)
Retinal pigment tear	3 (1.2)	2 (0.8)
Macular thickening	1 (0.4)	0 (0.0)
Visual impairment	1 (0.4)	2 (0.8)
Vitreoretinal traction syndrome	1 (0.4)	0 (0.0)
Age-related macular degeneration	0 (0.0)	1 (0.4)
Eye inflammation	0 (0.0)	1 (0.4)
Iritis	0 (0.0)	1 (0.4)
Macular hole	0 (0.0)	1 (0.4)
Macular edema	0 (0.0)	1 (0.4)
Ocular hypertension	0 (0.0)	1 (0.4)
Retinal vasculitis	0 (0.0)	1 (0.4)
Vitreous floaters	0 (0.0)	1 (0.4)

Ref-AFL = reference aflibercept; SAE = serious adverse event; SDZ-AFL = Sandoz aflibercept; TEAE = treatment-emergent adverse event.

The proportion of patients who were positive for ADAs at baseline (pre-treatment) was comparable between the treatment groups (1.2% and 1.7% in the SDZ-AFL and Ref-AFL groups, respectively) in the Mylight study;^36^ these data align with the incidence of ADAs reported in previous studies with aflibercept 2 and 8 mg (1%–3%^6^ and 2.5%–4.4%^5^). All samples that were ADA-positive at baseline (pre-treatment) were non-neutralizing. Over the 52-week study, the ADA incidence was found to be low: 0.9% and 2.6% in the SDZ-AFL and Ref-AFL groups, respectively. All ADA-positive patients developed neutralizing ADAs; most samples were titer-negative or low-titer (1:10).^36^

PK assessments revealed that systemic concentrations of free aflibercept 24 hours post-dose were both low and comparable between treatment groups.^36^ At day 2, the mean SDZ-AFL concentration was 32.0 ng/mL (range: 0–91.2; *n* = 21) and the mean Ref-AFL concentration was 33.3 ng/mL (range: 7.7– 93.4; *n* = 20). At day 58, the mean SDZ-AFL concentration was 31.7 ng/mL (range: 0–80.9; *n* = 22) and the mean Ref-AFL concentration was 33.6 ng/mL (range: 0–97.2; *n* = 17). The concentration of free VEGF in plasma was assessed in a subgroup of patients at week 48 (post-dose) and Week 52 (4 weeks post-dose). Systemic VEGF levels before dosing were comparable in the 2 treatment arms (SDZ-AFL: 86 pg/mL; Ref-AFL: 80 pg/mL). At 4 weeks post-dose, systemic VEGF levels had almost returned to pre-dose levels in both treatment arms (SDZ-AFL: 75 pg/mL; Ref-AFL: 73 pg/mL).^36^

#### In-Use Studies of SDZ-AFL

Participants received a single dose of SDZ-AFL provided in a vial kit (Study 303; *n* = 36)^37^ or a prefilled syringe (Study 304; *n* = 30)^38^ and were followed up for approximately 31 days.^37^^,^^38^ The reported adverse events for SDZ-AFL in these studies were in line with the established safety profile of Ref-AFL, indicating that the administration of SDZ-AFL provided in either a vial kit or a prefilled syringe has an acceptable safety profile.

## Discussion

The totality of evidence presented herein supports that the biosimilar SDZ-AFL is comparable to its reference biologic Ref-AFL in terms of in vitro physicochemical attributes and biological activity. The biosimilar matched the reference biologic in clinical safety, efficacy, immunogenicity, and PK outcomes. A comprehensive set of analytical studies utilizing state-of-art techniques revealed that SDZ-AFL and Ref-AFL have identical primary structures and comparable higher-order structures, along with comparable glycosylation variants, size variants, and charge variants, with minor justifiable differences. Biological activity was assessed using binding assays and cell-based functional assays, which showed that SDZ-AFL and Ref-AFL bind similarly to VEGF-A and inhibit VEGF-A-mediated downstream cell proliferation to a comparable degree.^39^ Compared with Ref-AFL, SDZ-AFL has a small but consistent increase in potency, which was attributed to lower levels of product-related impurities and determined not to be clinically relevant.

A non-clinical PK study in rabbits further supports these analytical data by demonstrating comparable vitreal distribution and elimination between SDZ-AFL and Ref-AFL. As reported previously, the pivotal Phase III Mylight study (NCT04864834) in patients with nAMD confirmed clinical equivalence between SDZ-AFL and Ref-AFL. It showed both treatments to have comparable safety, immunogenicity, and systemic PK, with no new safety signals detected.^36^ This study met its primary endpoint, demonstrating clinical equivalence between biosimilar and reference aflibercept concerning the mean change in BCVA from baseline to week 8.^36^ Anatomical outcomes and the proportion of patients with intraretinal fluid at weeks 8 and 52 were also comparable between the treatment groups.^36^ In terms of immunogenicity, the incidence of ADAs was low and comparable between the treatment groups.^36^

Further clinical data were gathered from 2 in-use device studies,^37^^,^^38^ which indicated acceptable safety for SDZ-AFL provided in either a vial kit or a prefilled syringe. Among the 5 aflibercept biosimilars approved in the US by July 2025, 2 include a prefilled syringe as well as a vial kit.^40^ While prefilled syringes provide advantages in intravitreal injection over vials, their development is challenging for biosimilar manufacturers in terms of optimal design, sterile manufacturing, and patent restrictions.^41^

Following the review of this totality of evidence, the FDA^32^ and EMA^33^ approved SDZ-AFL as an aflibercept biosimilar in 2024. In the European Union, SDZ-AFL is approved for treating adults with nAMD, macular edema following RVO, DME, and myopic CNV.^33^ Currently, many US ophthalmologists treating retinal diseases opt to prescribe off-label bevacizumab rather than aflibercept because of its lower cost^42^ and despite the demonstrated efficacy and durability of response seen with aflibercept.^43^, ^44^, ^45^, ^46^ The introduction of aflibercept biosimilars into the market may help to reduce the pressure on healthcare systems and in turn improve patient access to this well-established treatment option.

The European Medicines Agency stated that an approved biosimilar is in general interchangeable with its reference biologic, which also applies to SDZ-AFL.^47^ In the US, FDA can approve a biosimilar also as an interchangeable product to allow substitution for the reference biologic without the need for the intervention of the prescribing health provider.^16^ For SDZ-AFL, this interchangeability designation has not yet been granted due to the pending exclusivity of competitors.^48^ Nevertheless, the FDA deemed SDZ-AFL interchangeable with its reference medicine^48^, based on the totality of evidence for SDZ-AFL as presented here. In addition, this comprehensive biosimilar data package is a prerequisite for biosimilar approval in those indications that have not been clinically studied, given the same mechanism of action of the biosimilar in the investigated and extrapolated indications.^49^

While the totality of evidence for SDZ-AFL has secured its approval by major regulatory agencies, it should be noted that the sample size for the PK study in rabbits was small, while the in-use studies utilized a single-arm, open-label design and included small study populations.^37^^,^^38^ These factors limit the generalizability of the results from these studies, but are counterbalanced by the large patient population size in the Phase III Mylight study,^36^ which provided sufficient exposure to the biosimilar and the reference product while detecting relevant safety signals, as is required in biosimilar development programs.^50^ The Phase III data for SDZ-AFL was consistent with data for other aflibercept biosimilars that were included in a meta-analysis of randomized controlled trials comparing aflibercept biosimilars and reference aflibercept in patients with nAMD.^51^

Nevertheless, the impact of daily practice use of aflibercept biosimilars in various anti-VEGF markets remains unknown. The absence of long-term safety and efficacy data is an issue faced by all recently approved medicines. However, collection of post-marketing surveillance data is expected for all biologics, including biosimilars.^50^ Moreover, the broader accumulation of real-world evidence also provides the opportunity for the assessment of biosimilars in studies that are not sponsor-funded. Thus far, their use across multiple disease areas for the past 2 decades has shown the efficacy and safety profiles of biosimilars in routine practice to be generally consistent with their reference biologics.^52^ In the coming years, real-world experience with SDZ-AFL will be carefully monitored, and state-of-the-art techniques will be used to assess its long-term safety and efficacy as well as cost-effectiveness.

## Conclusions

The comprehensive totality of evidence gathered for biosimilar aflibercept SDZ-AFL during its development demonstrated its structural, functional, and clinical comparability to reference aflibercept. Following the upcoming entry of SDZ-AFL into the market, robust real-world data are expected to confirm its long-term efficacy and safety. This data will also elucidate the pharmacoeconomic impact of aflibercept biosimilars on the treatment landscape for neovascular retinal diseases.

## Declaration of competing interest

The authors declare the following financial interests/personal relationships which may be considered as potential competing interests: The presented research was sponsored by Sandoz. CW, DU, FD, JH, LA, and PA are employees of Sandoz AG or affiliates. RS has received consultancy funding from AbbVie, Bayer, Roche, and Théa Laboratories.

## References

[1] Adams B.S., Sorhaitz W., Stringham J. (2025). StatPearls.

[2] Gale R.P., Mahmood S., Devonport H. (2019).

[3] Schmidt-Erfurth U., Kaiser P.K., Korobelnik J.F. (2014). Intravitreal aflibercept injection for neovascular age-related macular degeneration: ninety-six-week results of the VIEW studies. Ophthalmology.

[4] Heier J.S., Brown D.M., Chong V. (2012). Intravitreal aflibercept (VEGF trap-eye) in wet age-related macular degeneration. Ophthalmology.

[5] European Medicines Agency. *Eylea Summary of product characteristics*. https://www.ema.europa.eu/en/documents/product-information/eylea-epar-product-information_en.pdf. Accessed August 5, 2025.

[6] US Food and Drug Administration. *Eylea Product Information*. https://www.regeneron.com/downloads/EYLEA_FPI.pdf. Accessed August 5, 2025.

[7] Helotera H., Kaarniranta K. (2022). A linkage between angiogenesis and inflammation in neovascular age-related macular degeneration. Cells.

[8] Anguita R., Tasiopoulou A., Shahid S., Roth J., Sim S.Y., Patel P.J. (2021). Correction to: a review of aflibercept treatment for macular disease. Ophthalmol Ther.

[9] Anguita R., Tasiopoulou A., Shahid S., Roth J., Sim S.Y., Patel P.J. (2021). A review of aflibercept treatment for macular disease. Ophthalmol Ther.

[10] World Health Organization. *Blindness and vision impairment*. https://www.who.int/news-room/fact-sheets/detail/blindness-and-visual-impairment. Accessed August 5, 2025.

[11] Schmidt-Erfurth U., Chong V., Loewenstein A. (2014). Guidelines for the management of neovascular age-related macular degeneration by the European Society of Retina Specialists (EURETINA). Br J Ophthalmol.

[12] Kaiser P.K., Schmitz-Valckenberg M.S., Holz F.G. (2022). Anti-vascular endothelial growth factor biosimilars in ophthalmology. Retina.

[13] Campochiaro P.A. (2021). Retinal and choroidal vascular diseases: past, present, and future: the 2021 Proctor Lecture. Invest Ophthalmol Vis Sci.

[14] Korva-Gurung I., Kubin A.M., Ohtonen P., Hautala N. (2023). Incidence and prevalence of neovascular age-related macular degeneration: 15-year epidemiological study in a population-based cohort in Finland. Ann Med.

[15] Li Q., Wang M., Li X., Shao Y. (2023). Aging and diabetic retinopathy: inherently intertwined pathophysiological processes. Exp Gerontol.

[16] US Food and Drug Administration. *Biosimilars - review and approval*. https://www.fda.gov/drugs/biosimilars/review-and-approval. Accessed August 5, 2025.

[17] Bressler N.M., Kaiser P.K., Do D.V. (2024). Biosimilars of anti-vascular endothelial growth factor for ophthalmic diseases: a review. Surv Ophthalmol.

[18] Blackstone E.A., Joseph P.F. (2013). The economics of biosimilars. Am Health Drug Benefits.

[19] Sunaga T., Maeda M., Saulle R. (2024). Anti-vascular endothelial growth factor biosimilars for neovascular age-related macular degeneration. Cochrane Database Syst Rev.

[20] The Center for Biosimilars. *First ophthalmology biosimilar launches in US*. https://www.centerforbiosimilars.com/view/first-lucentis-biosimilar-launches-in-us. Accessed August 5, 2025.

[21] GaBi Online. *EMA recommends approval of ranibizumab, rituximab and trastuzumab biosimilars*. https://gabionline.net/biosimilars/news/ema-recommends-approval-of-ranibizumab-rituximab-and-trastuzumab-biosimilars. Accessed August 5, 2025.

[22] GaBi Online. *Biosimilars approved in the US*. https://www.gabionline.net/biosimilars/general/biosimilars-approved-in-the-us. Accessed August 5, 2025.

[23] Sharma S., Khan M.A., Chaturvedi A. (2019). Real-life clinical effectiveness of Razumab® (the world's first biosimilar of ranibizumab) in retinal vein occlusion: a subgroup analysis of the pooled retrospective RE-ENACT study. Ophthalmologica.

[24] Chakraborty D., Stewart M.W., Sheth J.U. (2021). Real-world safety outcomes of intravitreal ranibizumab biosimilar (razumab) therapy for chorioretinal diseases. Ophthalmol Ther.

[25] Balciuniene V.J., Butkute E., Dailide G., Varoniukaite A. (2024). https://abstracts.euretina.org/2024/lb24-164-2042/r/rechkPpTTMYinMdF1.

[26] Finger R., Nguyen T.H., Hatfield N., Addison J. (2024). https://abstracts.euretina.org/2024/ca24-2208-3191/r/recsDVqs0DCp8KF8K.

[27] Deligiannidis A., Caride S.G., Vega P.S. (2024).

[28] Ratra D., Roy K., Giridhar S., Madaan S. (2022). Comparison between ranibizumab biosimilar, innovator ranibizumab and bevacizumab in a real-world situation. Ophthalmol Ther.

[29] Yanagi Y., Takahashi K., Iida T. (2023). Cost-effectiveness analysis of ranibizumab biosimilar for neovascular age-related macular degeneration in Japan. Ophthalmol Ther.

[30] Yanagi Y., Takahashi K., Iida T. (2024). Cost-effectiveness analysis of ranibizumab biosimilar for neovascular age-related macular degeneration and its subtypes from the societal and patient perspectives in Japan. Ophthalmol Ther.

[31] Sharma A., Woo S.J., Kuppermann B.D. (2025). Aflibercept biosimilars - so near, yet so far. Expert Opin Biol Ther.

[32] US Food and Drug Administration (2024). Enzeevu prescribing information. https://www.accessdata.fda.gov/drugsatfda_docs/label/2024/761382s000lbl.pdf.

[33] European Medicines Agency. *Afqlir summary of product characteristics*. https://www.ema.europa.eu/en/documents/product-information/afqlir-epar-product-information_en.pdf. Accessed August 5, 2025.

[34] US Food and Drug Administration. *Scientific considerations in demonstrating biosimilarity to a reference product. Guidance for Industry*. https://www.fda.gov/media/82647/download. Accessed August 5, 2025.

[35] ClinicalTrials.gov. *NCT04864834: phase III study assessing the efficacy, safety and immunogenicity of SOK583A1 versus Eylea® in patients with neovascular age-related macular degeneration (Mylight)*. https://clinicaltrials.gov/study/NCT04864834. Accessed August 5, 2025.

[36] Bordon A.F., Kaiser P.K., Wolf A. (2024). Efficacy and safety of the proposed biosimilar aflibercept, SDZ-AFL, in patients with neovascular age-related macular degeneration: 52-week results from the phase 3 Mylight study. Retina.

[37] ClinicalTrials.gov. *NCT05282004: study of the safety of use of intravitreal SOK583A1 provided in a vial kit*. https://clinicaltrials.gov/study/NCT05282004. Accessed August 5, 2025.

[38] ClinicalTrials.gov. *NCT05161806: study of the safety of use of intravitreal SOK583A1 provided in a prefilled syringe*. https://clinicaltrials.gov/study/NCT05161806. Accessed August 5, 2025.

[39] Latifi-Navid H., Soheili Z.S., Samiei S. (2021). Network analysis and the impact of aflibercept on specific mediators of angiogenesis in HUVEC cells. J Cell Mol Med.

[40] US Food and Drug Administration. *Purple book database of licensed biological products - Eylea*. https://purplebooksearch.fda.gov/results?query=aflibercept&title=Eylea. Accessed August 5, 2025.

[41] Sharma A., Loewenstein A., Parachuri N. (2024). Biosimilar anti-VEGF - is prefilled syringe (PFS) a challenge?. Eye (Lond).

[42] Dickson S.R., James K.E. (2023). Medicare Part B spending on macular degeneration treatments associated with manufacturer payments to ophthalmologists. JAMA Health Forum.

[43] Quist S.W., Nab H., Postma M., Amarakoon S., van Asten F., Freriks R. (2025). A cost-minimization analysis of anti-VEGFs for the treatment of neovascular age-related macular degeneration in the Netherlands. Graefes Arch Clin Exp Ophthalmol.

[44] Virgili G., Curran K., Lucenteforte E., Peto T., Parravano M. (2023). Anti-vascular endothelial growth factor for diabetic macular oedema: a network meta-analysis. Cochrane Database Syst Rev.

[45] Watkins C., Paulo T., Buhrer C., Holekamp N.M., Bagijn M. (2024). Correction to: comparative efficacy, durability and safety of faricimab in the treatment of diabetic macular edema: a systematic literature review and network meta-analysis. Adv Ther.

[46] Watkins C., Paulo T., Buhrer C., Holekamp N.M., Bagijn M. (2023). Comparative efficacy, durability and safety of faricimab in the treatment of diabetic macular edema: a systematic literature review and network meta-analysis. Adv Ther.

[47] European Medicines Agency. *Statement on the scientific rationale supporting interchangeability of biosimilar medicines in the EU*. https://www.ema.europa.eu/en/documents/public-statement/statement-scientific-rationale-supporting-interchangeability-biosimilar-medicines-eu_en.pdf. Accessed August 5, 2025. 10.1136/ejhpharm-2022-003571 PMC9614114 36283720

[48] Sandoz. *Sandoz receives FDA approval for Enzeevu™ (aflibercept-abzv), further strengthening US biosimilar position*. https://www.sandoz.com/sandoz-receives-fda-approval-enzeevutm-aflibercept-abzv-further-strengthening-us-biosimilar/. Accessed August 5, 2025.

[49] Tesser J.R., Furst D.E., Jacobs I. (2017). Biosimilars and the extrapolation of indications for inflammatory conditions. Biologics.

[50] Sharma A., Kuppermann B.D. (2022). Biosimilars for retinal diseases: understanding the phase 3 clinical trial design. Ophthalmology.

[51] Aljuhani H.S., Hubayni R.A., Qedair J. (2025). Efficacy and safety of aflibercept biosimilars relative to reference aflibercept therapy for neovascular age-related macular degeneration: a systematic review and meta-analysis. Clin Ophthalmol.

[52] Sagi S., Anjaneya P., Kalsekar S., Kottke A., Cohen H.P. (2023). Long-term real-world post-approval safety data of multiple biosimilars from one marketing-authorization holder after more than 18 years since their first biosimilar launch. Drug Saf.

